# Adjunctive Short‐Wave Diathermy to High‐Frequency Chest Wall Oscillation for Acute Exacerbation of COPD: A Randomized Controlled Trial

**DOI:** 10.1155/carj/6813132

**Published:** 2026-09-18

**Authors:** Qinqin Li, Jielu Sun, Lujing Ying, Zaishuang Ying

**Affiliations:** ^1^ Yongkang First People’s Hospital, Jinhua, Zhejiang, China

**Keywords:** acute exacerbation chronic obstructive pulmonary disease, airway clearance, high-frequency chest wall oscillation, short-wave diathermy

## Abstract

**Objective:**

This study aimed to evaluate the efficacy and safety of combining short‐wave diathermy (SWD) with high‐frequency chest wall oscillation (HFCWO) compared with HFCWO alone and routine treatment in patients hospitalized for acute exacerbation of chronic obstructive pulmonary disease (AECOPD).

**Methods:**

This single‐center, parallel‐group randomized controlled trial enrolled 89 patients with AECOPD admitted to Yongkang First People’s Hospital between March 2024 and March 2025. Participants were randomly assigned (1:1:1) to receive routine treatment alone (control group), routine treatment plus HFCWO (HFCWO group), or routine treatment plus combined SWD and HFCWO (combined group) for 72 h. Primary and secondary outcomes were assessed at baseline and after 72 h of intervention, with both within‐group and between‐group comparisons performed. Primary outcomes included changes in the COPD Assessment Test (CAT) and Breathlessness, Cough, and Sputum Scale (BCSS) scores. Secondary outcomes included lung function parameters (FEV_1_, FVC, and FEV_1_/FVC), arterial blood gas parameters (PaO_2_ and PaCO_2_), inflammatory markers (interleukin‐6, C‐reactive protein, and white blood cell count), and quantitative sputum indices (24‐h sputum volume, total cell count, and differential counts).

**Results:**

After the 72‐h intervention, the combined therapy group demonstrated significantly greater reductions in CAT and BCSS scores compared with both the HFCWO group and the control group (*p* < 0.05). Combined therapy also achieved superior improvements in arterial blood gas parameters (PaO_2_ and PaCO_2_) and sputum clearance indices (24‐h sputum volume and neutrophil and eosinophil percentages) compared with HFCWO alone or routine treatment. In contrast, no significant differences were observed in lung function parameters (FEV_1_ and FVC) or inflammatory markers (IL‐6, CRP, and WBC).

**Conclusions:**

The combined intervention significantly improves respiratory symptoms, arterial oxygenation, and sputum clearance in patients with AECOPD compared with HFCWO alone or routine treatment. The addition of thermal therapy enhances the clinical efficacy of mechanical airway clearance, offering a feasible adjunctive strategy for acute‐phase management of AECOPD.

## 1. Introduction

Acute exacerbation of chronic obstructive pulmonary disease (AECOPD), characterized by acute deterioration of respiratory symptoms beyond day‐to‐day variability, represents a critical event in the natural history of the disease [1, 2]. According to the 2024 Global Initiative for Chronic Obstructive Lung Disease (GOLD) guidelines, current standard management for infective AECOPD includes controlled oxygen therapy, short‐acting bronchodilators, and antibiotics [3]. Despite standard pharmacotherapy, persistent sputum retention and airway obstruction still drive lung function decline, bacterial colonization, longer hospital stays, and higher medical costs [4, 5]. Consequently, adjunctive physical therapies aimed at enhancing mucus clearance and improving quality of life have gained increasing attention as integral components of comprehensive AECOPD management.

Currently, high‐frequency chest wall oscillation (HFCWO) represents one of the principal physical therapeutic modalities for enhancing airway clearance in patients with respiratory diseases [6]. This technique employs an inflatable vest to generate oscillatory compressions at frequencies of 12–15 Hz against the chest wall, transmitting vibrational energy to the airways to dislodge mucus from the bronchial walls and facilitating cephalad movement through pulsatile airflow [7]. Although HFCWO has demonstrated efficacy in cystic fibrosis and bronchiectasis, its reliance on mechanical vibration alone may be insufficient to effectively clear the characteristically viscous, tenacious sputum observed in AECOPD patients [8]. Short‐wave diathermy (SWD) offers a mechanistically complementary approach, utilizing high‐frequency electromagnetic energy to produce deep tissue thermal effects that reduce sputum viscosity, improve local blood circulation, enhance mucociliary transport function, and potentially increase the compliance of retained secretions [9, 10]. Theoretically, the sequential combination of SWD and HFCWO constitutes a synergistic strategy wherein pretreatment thermal conditioning reduces sputum tenacity and improves rheological properties, thereby facilitating subsequent mechanical loosening and evacuation by HFCWO. Nevertheless, this combined therapy has not been rigorously evaluated in randomized controlled trials, and existing physical therapy studies lack objective quantitative sputum indices, dynamic monitoring of systemic inflammatory markers, and assessment of combined treatment modalities. Consequently, clinicians lack clear guidance regarding whether adjunctive physical therapies—particularly combined thermal and mechanical interventions—provide significant incremental benefit over standard pharmacological treatment in acute settings.

This study aims to evaluate the efficacy and safety of combined SWD and HFCWO versus HFCWO alone and routine treatment alone in symptom relief, lung function improvement, and sputum clearance among patients with AECOPD through a single‐center, randomized controlled trial. The findings of this study will provide evidence‐based guidance for the selection of physical therapy during the acute phase of AECOPD and explore the clinical application value of combined thermal and mechanical intervention.

## 2. Study Design and Methods

### 2.1. Design

This was a single‐center, parallel‐group randomized controlled trial conducted at Yongkang First People’s Hospital from March 2024 to March 2025. Eligible patients with AECOPD were randomly assigned in a 1:1:1 ratio to receive either HFCWO alone, SWD combined with HFCWO, or routine treatment alone. Randomization was performed using a computer‐generated random sequence (https://www.jyshare.com/front-end/6680) with allocation concealment achieved through sequentially numbered, opaque, sealed envelopes. Due to the nature of physical interventions, blinding of participants and treating physicians were not feasible; however, outcome assessors and data analysts remained blinded to group allocation throughout the study. Based on the treatment window for AECOPD patients and the immediate physiological effects of airway clearance techniques, the intervention period was set at 72 h (3 days), with assessments conducted at baseline and Day 3. This study was conducted in accordance with the Declaration of Helsinki and approved by the Ethics Committee of Yongkang First People’s Hospital (Approval Number: YKSDYRMYYEC2024‐KT‐HS‐024‐02). Written informed consent was obtained from all participants.

### 2.2. Subjects

The sample size was estimated a priori based on the primary outcome of change in the COPD Assessment Test (CAT) score from baseline to 72 h. A pilot study conducted at our center prior to the formal trial provided preliminary data showing mean reductions of 1.5 points in the control group, 4.0 points in the HFCWO group, and 6.5 points in the combined group, with a pooled standard deviation of 6.0 points. Using one‐way analysis of variance (ANOVA) for three independent groups (G∗Power 3.1.9.7; Heinrich‐Heine‐Universität Düsseldorf, Germany), with a two‐sided significance level of 0.05 and a statistical power of 0.80, the minimum required total sample size was 84 patients (28 per group). With an anticipated dropout rate of 10%, the target enrollment was set at 93 patients. In total, 99 patients were randomized and completed the intervention, meeting the planned sample size.

In total, 89 patients hospitalized with AECOPD were recruited from the Department of Respiratory Medicine and Emergency Department at Yongkang First People’s Hospital between March 2024 and March 2025. Infective acute exacerbation was defined as worsening dyspnea, increased cough and sputum production, changes in sputum color or consistency, and fever, accompanied by radiographic evidence of pulmonary consolidation.

Inclusion criteria are as follows: (1) meeting GOLD 2024 AECOPD diagnostic criteria, acute worsening of dyspnea, cough, or sputum beyond day‐to‐day variability within 14 days, requiring treatment adjustment, accompanied by purulent sputum or radiographic evidence of pulmonary infection and GOLD Grades 3–4; (2) aged 50–80 years, male or female; (3) conscious, capable of cooperating with scale assessment and pulmonary function test, and signed informed consent; and (4) no systemic corticosteroids, mucolytic agents, or physical therapy within 2 weeks prior to enrollment.

Exclusion criteria are as follows: (1) concomitant pneumothorax, pulmonary embolism, acute heart failure, or pulmonary encephalopathy requiring emergency resuscitation; (2) contraindications to physical therapy, including thoracic metallic implants, cutaneous lesions or infection, thoracic cage deformity, or recent thoracic surgery (within 3 months); (3) coexisting bronchiectasis, active pulmonary tuberculosis, lung cancer, or other parenchymal lung diseases; (4) severe hepatic or renal insufficiency, active bleeding, or coagulation disorders; (5) pregnancy or lactation, or participation in another interventional clinical trial within the preceding 30 days; and (6) severe cognitive impairment, psychiatric disorders, or language barriers precluding adequate assessment.

### 2.3. Intervention

All enrolled patients received routine treatment for AECOPD according to the 2024 Global Initiative for GOLD guidelines. Treatment measures included controlled oxygen therapy, short‐acting bronchodilators (salbutamol 2.5 mg combined with ipratropium bromide 500 μg via nebulization every 6–8 h), systemic corticosteroids (prednisone equivalent 1 mg/kg/day, maximum 60 mg/day for 3 days), antibiotics, and health education. Participants were randomly assigned in a 1:1:1 ratio to three parallel groups: The control group received routine treatment alone; the HFCWO group received routine treatment plus HFCWO; and the combined therapy group received routine treatment plus HFCWO and SWD.

HFCWO was performed using an inflatable vest (PT‐200HM, Henan Maitong Industrial Co., Ltd., China) wrapped around the patient’s chest wall. The device oscillation frequency was set at 10–14 Hz, and oscillation intensity was adjusted according to individual patient tolerance. Each treatment session lasted 20 min, twice daily (morning and evening, with an interval of ≥ 4 h between sessions), for 3 consecutive days. During treatment, patients were placed in a semi‐reclining position (head‐of‐bed elevation 30°–45°) and were instructed to perform huff coughing every 5 min. SWD was delivered using a SWD unit (DL‐C‐M, Shantou Medical Equipment Factory Co., Ltd., China). Capacitive electrodes were placed on the patient’s thoracic back (T3–T10 vertebral levels). Treatment parameters were set to continuous mode, with an initial power of 50 W, gradually increased to patient‐tolerable warm sensation according to individual tolerance. An infrared thermometer (ThermoScan 7, Braun GmbH, Germany) was used to monitor skin temperature, maintaining skin temperature ≤ 41°C. Each SWD session lasted 20 min, once daily, performed immediately before HFCWO, for 3 consecutive days. Electrode‐to‐skin distance was maintained at 3–5 cm during treatment, and petroleum jelly was applied to the skin surface to prevent burns.

### 2.4. Outcome Measurements

The primary endpoint was change in patient symptom scores, assessed using the CAT and the Breathlessness, Cough, and Sputum Scale (BCSS). The CAT scale is used to assess health‐related quality of life in COPD patients, covering eight dimensions including cough, sputum, chest tightness, breathlessness, sleep, energy, emotion, and exercise tolerance, with a total score of 0–40 points, where 0 indicates optimal health status and higher scores indicate worse quality of life. The BCSS is used to assess the severity of core respiratory symptoms in COPD patients, including three items: breathlessness, cough, and sputum, each scored 0–4 according to severity, with a total score of 0–12 points, where higher scores indicate more severe symptoms. Both scales were administered by trained assessors at enrollment (baseline) and on Day 3 of treatment, using standardized instructions to ensure scoring consistency.

Secondary endpoints included (1) lung function indices: forced expiratory volume in 1 s (FEV_1_), forced vital capacity (FVC), and FEV_1_/FVC ratio; (2) blood gas analysis indices: arterial partial pressure of oxygen (PaO_2_) and arterial partial pressure of carbon dioxide (PaCO_2_); (3) inflammatory indices: interleukin‐6 (IL‐6), C‐reactive protein (CRP), and white blood cell count (WBC); and (4) sputum indices: 24‐h sputum volume (MSV), total sputum cell count (TCC), and differential cell counts (neutrophil percentage [NEU], lymphocyte percentage [LYM], and eosinophil percentage [EOS]). Pulmonary function and blood gas analysis were performed by professional technicians. IL‐6 was detected by enzyme‐linked immunosorbent assay. CRP, WBC, and blood cell differential counts were measured by an automated hematology analyzer. Sputum was collected through spontaneous expectoration over a 24‐h period. Sputum wet weight was measured by an electronic scale. Sputum was centrifuged, and sediment was taken for total cell count and differential count. The counting process was completed by laboratory technicians who were unaware of group allocation. All indices were collected and measured at baseline and on Day 3 after treatment. Collection time was 8:00–10:00 (AM) to avoid circadian rhythm influence.

### 2.5. Statistical Analysis

Normality of quantitative variables was assessed by the Shapiro–Wilk test. Normally distributed data were expressed as mean ± standard deviation, nonnormally distributed data were expressed as median (interquartile range [IQR]), and categorical variables were described as absolute numbers and relative frequencies. Comparisons between two groups and among three groups for normally distributed data were performed using *t*‐test and ANOVA, respectively. Nonnormally distributed data were analyzed using the Kruskal–Wallis test. Categorical data were compared using the chi‐square test. Statistical analysis was performed using SPSS software (version 22, IBM Corp., Armonk, NY, USA). Median values of CAT and BCSSs were compared and plotted using GraphPad Prism software (version 9.0, GraphPad Software, USA). The significance level α was set at 0.05.

## 3. Results

### 3.1. Descriptive Analysis

**FIGURE 1:**
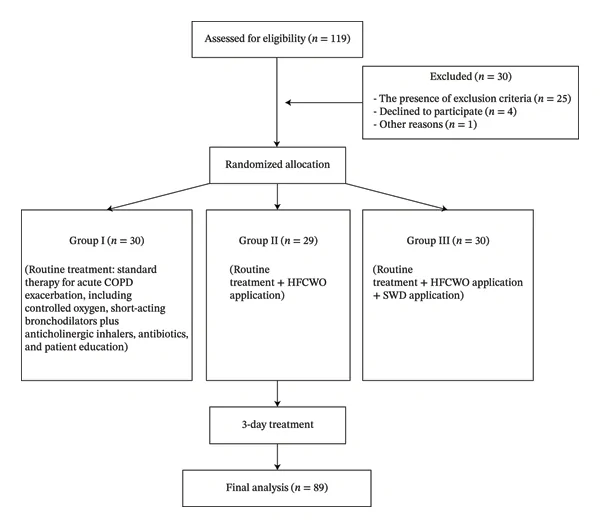
Flow chart detailing study methodology.

A total of 129 patients were screened for eligibility, of whom 25 were excluded due to meeting exclusion criteria, 4 declined to participate, and 1 was excluded for other reasons, leaving 89 patients who met the inclusion criteria. The study flow chart and reasons for exclusion are presented in Figure 1. The final study sample comprised 99 patients randomly assigned to Group I (routine treatment, *n* = 30), Group II (routine treatment + HFCWO, *n* = 29), and Group III (routine treatment + HFCWO + SWD, *n* = 30). Table 1 presents the baseline demographic, clinical, functional, and inflammatory characteristics of patients in the control, HFCWO, and HFCWO + SWD groups. No statistically significant differences were found among the three groups in any baseline parameters (all *p* > 0.05), indicating good baseline comparability.

**TABLE 1:** Baseline demographic, clinical, functional, and inflammatory characteristics of participants in the three groups.

Variable	Control group (*N* = 30)	HFCWO group (*N* = 29)	HFCWO + SWD group (*N* = 30)	*p*‐Value
Demographic characteristics				
Male (%)	14 (46.67%)	16 (53.33%)	16 (53.33%)	0.871
Age (years)	67.54 ± 3.25	66.42 ± 3.49	67.18 ± 3.74	0.810
BMI (kg/m^2^)	25.62 ± 3.81	25.84 ± 3.76	25.18 ± 3.69	0.793
Scales				
CAT	20.25 [11.00, 30.50]	20.50 [11.50, 31.00]	21.00 [11.00, 32.00]	0.749
BCSS	6.00 [3.50, 9.50]	6.50 [3.00, 9.00]	6.00 [3.00, 9.00]	0.889
Lung function				
FEV_1_ (L)	0.76 ± 0.25	0.81 ± 0.31	0.83 ± 0.39	0.245
FVC (L)	1.52 ± 0.39	1.42 ± 0.67	1.45 ± 0.59	0.298
FEV_1_/FVC	48.62 ± 11.38	49.71 ± 13.69	49.09 ± 12.74	0.642
Inflammatory indicators				
IL‐6 (pg/mL)	191.27 ± 30.44	186.80 ± 35.36	181.62 ± 32.96	0.113
CRP (mg/L)	21.52 ± 7.21	22.54 ± 6.93	23.10 ± 7.34	0.166
WBC (× 10^9^/L)	11.77 ± 3.23	11.45 ± 3.38	10.89 ± 3.47	0.477
Sputum biomarkers				
MSV (mL/24 h)	47.25 ± 8.63	49.34 ± 9.65	50.08 ± 9.71	0.264
TCC (× 10^6^/g)	4.81 ± 0.93	4.56 ± 1.02	4.61 ± 0.86	0.209
NEU (%)	59.34 ± 10.21	58.14 ± 9.85	61.62 ± 11.19	0.335
LYM (%)	2.82 ± 0.33	2.75 ± 0.42	2.62 ± 0.46	0.237
EOS (%)	2.68 ± 0.49	2.72 ± 0.51	2.63 ± 0.57	0.504
Blood gas indicators				
PaO_2_ (mm Hg)	71.36 ± 12.32	72.84 ± 11.78	69.84 ± 13.40	0.318
PaCO_2_ (mm Hg)	40.55 ± 7.28	41.09 ± 8.26	42.17 ± 8.34	0.392

*Note:* Data are expressed as mean ± SD, median (IQR), or *n* (%). *p*‐value < 0.05; F‐test for continuous variables and *χ* ^2^ for categorical variables.

Abbreviations: BCSS, Breathlessness, Cough, and Sputum Scale; CAT, COPD Assessment Test; CRP, C‐reactive protein; EOS, eosinophil percentage; FEV1, forced expiratory volume in one second; FEV1/FVC, forced expiratory volume in one second/forced vital capacity; FVC, forced vital capacity; IL‐6, interleukin‐6; LYM, lymphocyte percentage; MSV, mucoid sputum volume; NEU, neutrophil percentage; PaCO_2_, partial pressure of arterial carbon dioxide; PaO_2_, partial pressure of arterial oxygen; TCC, total cell count; WBC, white blood cell count.

### 3.2. Primary Outcomes

**FIGURE 2:**
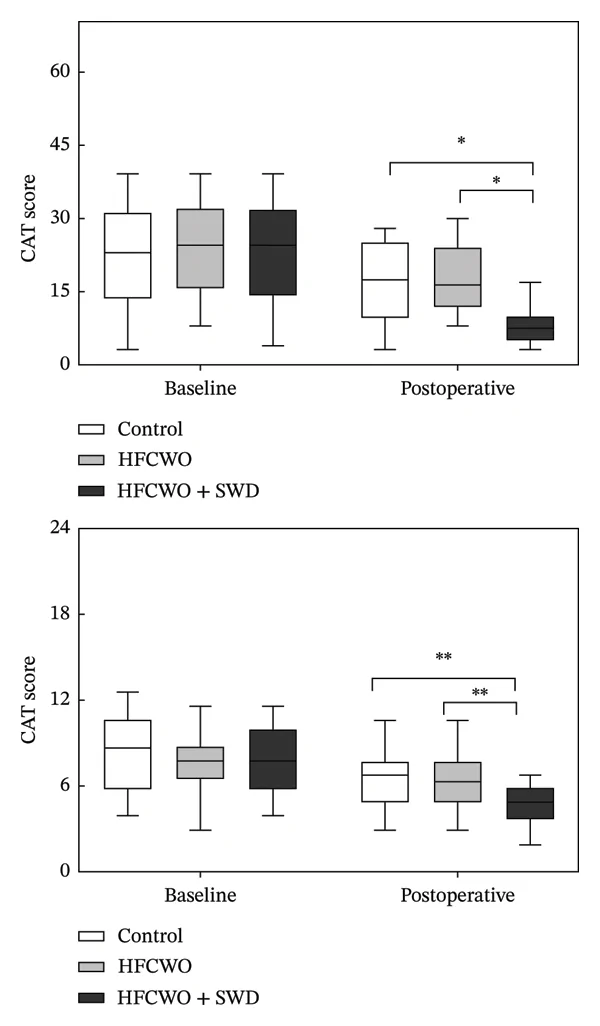
Median changes in CAT and BCSS scores from baseline to Day 3 across the three groups.

The Kruskal–Wallis test was used to compare CAT and BCSS scores among the three groups. No significant differences were observed at baseline (all *p* > 0.05). After the 72‐h intervention, scores decreased from baseline in all three groups. The median CAT scores were 18.0 in the control group, 17.5 in the HFCWO group, and 7.0 in the combined group; the median BCSS scores were 7.0, 6.5, and 4.0, respectively. The Kruskal–Wallis test revealed significant differences among the three groups for both CAT and BCSS (*p* < 0.001). Pairwise comparisons showed that the combined group had significantly lower CAT and BCSS scores than both the control group and the HFCWO group (all adjusted *p* < 0.05), whereas no significant difference was observed between the control group and the HFCWO group (adjusted *p* > 0.05). See Figure 2.

### 3.3. Secondary Outcomes

Compared with the control group, both treatment methods improved multiple patient indices (FEV_1_, FVC, FEV_1_/FVC, PaO_2_, PaCO_2_, MSV, TCC, NEU, and EOS). However, direct comparison between the HFCWO and HFCWO + SWD groups revealed significant improvements in the combined therapy group for blood gas parameters (PaO_2_ and PaCO_2_) and sputum indices (MSV, NEU, and EOS) (*p* < 0.05). These results are shown in Tables 2, 3, and 4, respectively. In addition, no treatment‐related adverse events were recorded during the 72‐h intervention period across all three groups. No participants presented SWD‐ or HFCWO‐associated discomfort including skin redness, burning sensation, chest pain, exacerbated dyspnea, dizziness, or nausea.

**TABLE 2:** Comparison of lung function and blood gas parameters before and after treatment in the three groups.

Variable	Before treatment			After treatment			*p*‐Value^†^		
	Control group	HFCWO group	HFCWO + SWD group	Control group	HFCWO group	HFCWO + SWD group	HFCWO/control	HFCWO + SWD/control	HFCWO + SWD/HFCWO
Pulmonary function									
FEV_1_ (L)	0.76 ± 0.25	0.75 ± 0.31	0.77 ± 0.39	0.87 ± 0.31^∗^	1.06 ± 0.28^∗^	1.04 ± 0.33^∗^	< 0.01	0.013	0.849
FVC (L)	1.52 ± 0.39	1.42 ± 0.67	1.45 ± 0.59	1.61 ± 0.41^∗^	1.79 ± 0.53^∗^	1.76 ± 0.42^∗^	0.020	< 0.01	0.755
FEV_1_/FVC	48.62 ± 11.38	49.71 ± 13.69	49.09 ± 12.74	54.27 ± 10.62^∗^	59.83 ± 11.79^∗^	60.62 ± 13.21^∗^	0.024	< 0.001	0.955
Blood gas indicators									
PaO_2_ (mm Hg)	68.36 ± 12.32	70.84 ± 11.78	69.84 ± 13.40	69.62 ± 9.68	74.75 ± 8.51^∗^	79.34 ± 9.94^∗^	< 0.001	< 0.001	0.028
PaCO_2_ (mm Hg)	40.55 ± 7.28	41.09 ± 8.26	42.17 ± 8.34	42.36 ± 8.20	37.63 ± 9.35^∗^	33.09 ± 8.64^∗^	< 0.001	< 0.001	0.032

Abbreviations: FEV_1_, forced expiratory volume in one second; FEV_1_/FVC, forced expiratory volume in one second/forced vital capacity; FVC, forced vital capacity.

^∗^Within‐group comparisons (before vs. after treatment) were statistically significant (*p* < 0.05).

^†^Posttreatment pairwise between‐group comparisons.

**TABLE 3:** Comparison of hematological and inflammatory parameters before and after treatment in the three groups.

Variable	Before treatment			After treatment			*p*‐Value^†^		
	Control group	HFCWO group	HFCWO + SWD group	Control group	HFCWO group	HFCWO + SWD group	HFCWO/control	HFCWO + SWD/control	HFCWO + SWD/HFCWO
IL‐6 (pg/mL)	191.27 ± 30.44	186.80 ± 35.36	181.62 ± 32.96	121.27 ± 30.15^∗^	117.18 ± 32.09^∗^	119.08 ± 33.13^∗^	0.296	0.337	0.561
CRP (mg/L)	21.52 ± 7.21	22.54 ± 6.93	23.10 ± 7.34	11.43 ± 2.90^∗^	11.09 ± 3.54^∗^	10.98 ± 2.85^∗^	0.868	0.759	0.801
WBC (× 10^9^/L)	11.77 ± 3.23	11.45 ± 3.38	10.89 ± 3.47	8.35 ± 2.42^∗^	8.49 ± 2.28^∗^	8.33 ± 2.93^∗^	0.905	0.963	0.924

Abbreviations: CRP, C‐reactive protein; IL‐6, interleukin‐6; WBC, white blood cell count.

^∗^Within‐group comparisons (before vs. after treatment) were statistically significant (*p* < 0.05).

^†^Posttreatment pairwise between‐group comparisons.

**TABLE 4:** Comparison of sputum volume and sputum cell count before and after treatment in the three groups.

Variable	Before treatment			After treatment			*p*‐Value^†^		
	Control group	HFCWO group	HFCWO + SWD group	Control group	HFCWO group	HFCWO + SWD group	HFCWO/control	HFCWO + SWD/control	HFCWO + SWD/HFCWO
MSV (mL/24 h)	4.81 ± 0.93	4.76 ± 1.02	4.69 ± 0.86	45.39 ± 8.96	61.08 ± 9.35^∗^	78.75 ± 10.74^∗^	< 0.001	< 0.001	< 0.001
TCC (× 10^6^/g)	69.34 ± 10.21	68.14 ± 9.85	71.62 ± 11.19	5.60 ± 0.89^∗^	4.74 ± 1.10	4.55 ± 1.16	< 0.01	< 0.01	0.870
NEU (%)	2.82 ± 0.33	2.75 ± 0.42	2.62 ± 0.46	64.61 ± 8.57^∗^	59.17 ± 9.18^∗^	54.36 ± 10.20^∗^	0.030	< 0.001	0.042
LYM (%)	2.68 ± 0.49	2.72 ± 0.51	2.63 ± 0.57	2.71 ± 0.64	2.69 ± 0.74	2.76 ± 0.67	0.859	0.774	0.793
EOS (%)	4.81 ± 0.93	4.76 ± 1.02	4.69 ± 0.86	2.60 ± 0.55	2.37 ± 0.49^∗^	1.98 ± 0.41^∗^	0.019	< 0.001	0.008

Abbreviations: EOS, eosinophil percentage; LYM, lymphocyte percentage; MSV, mucoid sputum volume; NEU, neutrophil percentage; TCC, total cell count.

^∗^Within‐group comparisons (before vs. after treatment) were statistically significant (*p* < 0.05).

^†^Posttreatment pairwise between‐group comparisons.

## 4. Discussion

The present randomized controlled trial investigated the efficacy of combining SWD with HFCWO in patients hospitalized with AECOPD. Our principal findings indicate that, compared with HFCWO alone or routine treatment, the combined intervention achieved significantly greater improvements in respiratory symptoms, arterial blood gas parameters, and quantitative sputum indices over a 72‐h treatment period. Specifically, patients receiving combined therapy demonstrated marked reductions in CAT and BCSS scores, accompanied by superior oxygenation, reduced hypercapnia, and enhanced neutrophilic sputum clearance. These results provide preliminary evidence that thermal preconditioning may augment the clinical effectiveness of mechanical airway clearance techniques in the management of AECOPD, offering a feasible adjunctive strategy to accelerate symptom resolution and mucus evacuation during the critical early phase of hospitalization.

The observed improvements in symptom scores hold significant clinical implications for the management of acute exacerbations. During infective exacerbations, substantially increased sputum viscosity may attenuate the transmission of vibrational energy from chest wall oscillation to distal airways. Previous studies have demonstrated suboptimal improvement in CAT scores following long‐term, low‐frequency interventions. For instance, Kotani reported that pulmonary rehabilitation over 2 years failed to significantly reduce CAT scores or alleviate patient symptoms [11]. The present study found that the combined therapy group exhibited greater reductions in both CAT and BCSS scores compared with those receiving HFCWO alone or routine treatment, suggesting that thermal preconditioning may effectively address the limitations of oscillation therapy alone. This discrepancy likely stems from the fact that single‐modality mechanical intervention relies primarily on passive vibration without altering sputum rheological properties, whereas SWD‐induced thermal effects actively reduce mucus viscosity and enhance the compliance of retained secretions prior to mechanical clearance [12]. The BCSS specifically targets the three cardinal symptoms of acute exacerbation—dyspnea, cough, and sputum production—rendering its score improvements directly relevant to patient‐centered outcomes [13, 14]. For example, a meta‐analysis by Yang found that HFCWO enabled patients with AECOPD to expectorate more sputum and alleviate symptoms [15]. However, this finding requires further validation due to heterogeneity among included studies. Nicolini et al. demonstrated that HFCWO significantly improved CAT and BCSS scores following 4 weeks of treatment. By contrast, the combined therapy group in the present study achieved significant reductions in BCSS scores within 72 h, with a greater magnitude of improvement, highlighting the rapid onset and synergistic effects of the integrated approach [16]. Oscillation therapy alone cannot directly facilitate mechanical sputum evacuation and immediately alleviate the symptomatic burden of greatest concern to patients. This further underscores the superior clinical value of combined therapy in acute management, as it concurrently addresses both inflammatory control (through standard pharmacotherapy) and physical retention of secretions (through HFCWO‐mediated mechanical clearance).

In addition to symptomatic improvement, combined therapy significantly ameliorated arterial blood gas parameters. The SWD + HFCWO group exhibited elevated PaO_2_ and reduced PaCO_2_ compared with both the control and HFCWO‐only groups, suggesting that synergistic clearance of retained secretions facilitated more efficient gas exchange, likely through restoration of ventilation–perfusion matching in previously obstructed airways [17]. This mechanism is supported by previous investigations; for example, Cheng et al. reported significant improvement in PaO_2_ following HFCWO therapy in patients with AECOPD, albeit with an intervention duration of 14 days [18]. The more rapid normalization of gas exchange observed in the present study (within 72 h) may reflect the additional thermal component accelerating sputum mobilization kinetics. Mechanical airway clearance can rapidly improve oxygenation in patients with acute respiratory disease by reducing intrapulmonary shunt [19]. However, improvements in pulmonary function assessed by FEV_1_ and FVC were modest, with no statistically significant differences among the three groups. Mahajan et al. similarly reported that HFCWO exerted no significant therapeutic effect on pulmonary function in patients with acute asthma or COPD, particularly regarding FEV_1_ [20]. This discordance between pulmonary function and gas exchange outcomes is consistent with the differential time course of physiological recovery in AECOPD. Previous longitudinal studies have demonstrated that the median time for pulmonary function recovery in patients with moderate‐to‐severe COPD extends to several weeks following treatment [21]. Consequently, the 3‐day observation window in the present study may have captured only the early gas exchange benefits conferred by mechanical clearance, whereas comprehensive recovery of pulmonary function requires a more extended time frame.

Combined therapy also exerted significant effects on quantitative sputum indices. Patients receiving SWD combined with HFCWO demonstrated significant increases in 24‐h MSV and significant reductions in NEU and EOS, indicating enhanced mucus evacuation from peripheral airways and attenuation of acute airway inflammation. These findings are consistent with previous investigations. For instance, Nielsen et al. reported that HFCWO and intrapulmonary percussive ventilation over 4 weeks resulted in decreased sputum neutrophil and macrophage counts, suggesting that effective airway clearance can reduce neutrophil burden in patients with chronic suppurative lung disease [22]. The more pronounced cellular response observed in the present study may reflect accelerated evacuation of bacteria‐laden mucus, thereby interrupting the inflammatory stimulus driving neutrophil recruitment. However, no significant differences were observed in TCC, which may indicate that the 72‐h observation window captured only the early effects of secretion clearance, whereas comprehensive changes in inflammatory cell composition within the airways require a more extended time frame to manifest [23]. The pathogenesis of AECOPD is complex, primarily involving viral or bacterial infection, airway inflammation, and mucus hypersecretion [24]. Notably, systemic inflammatory markers including IL‐6, CRP, and WBC improved from baseline across all three groups, yet no statistically significant differences were observed among the treatment groups. This may reflect the dominant anti‐inflammatory and antimicrobial effects of standardized pharmacotherapy (systemic corticosteroids and antibiotics) administered to all patients, which may have partially overshadowed the effects of physical intervention alone [25, 26].

This study has several limitations. First, the single‐center design may restrict the generalizability of our findings to populations with different regional characteristics, healthcare accessibility, and treatment resources. Second, the 72‐h intervention period, while sufficient to capture acute symptomatic and physiological responses, precludes assessment of sustained effects on pulmonary function recovery or long‐term survival outcomes. Third, we did not collect data on length of hospital stay, duration of antibiotic therapy, or total treatment costs, which limits our ability to evaluate the cost‐effectiveness of combined thermal–mechanical therapy. Future research should validate the external validity of these findings through multicenter randomized controlled trials incorporating larger, geographically diverse samples. Additionally, extended follow‐up periods are warranted to evaluate sustained symptomatic benefits and recovery of pulmonary function parameters.

## 5. Conclusions

This single‐center, 72‐h trial found that combined SWD and HFCWO produced greater improvements in respiratory symptoms, blood gas indices, and sputum clearance than HFCWO alone or routine treatment among hospitalized AECOPD patients. Within this 3‐day acute intervention window, adjunctive SWD appeared to boost the acute benefits of mechanical airway clearance. Nevertheless, these preliminary findings cannot be broadly generalized due to the single‐center design and short follow‐up. Future multicenter trials with extended observation are needed to confirm its generalizability. These trials should adopt a sham SWD control arm to mitigate placebo bias and strengthen study validity and also incorporate hospital length of stay and 30‐day readmission as secondary endpoints to assess clinical disease burden.

## Author Contributions

Qinqin Li designed the present study, obtained funding, reviewed the manuscript, and assisted with manuscript revision. Jielu Sun conducted data collection, performed the statistical analyses, and drafted and revised the manuscript. Lujing Ying, and Zaishuang Ying contributed to the data acquisition and final version approval.

## Funding

This work was supported by Project No. 2024‐4‐312: Observation on the efficacy of short‐wave irradiation combined with high‐frequency chest wall oscillation in patients with acute exacerbation of COPD.

## Disclosure

All authors read and approved the final manuscript.

## Conflicts of Interest

The authors declare no conflicts of interest.

## Data Availability

The data that support the findings of this study are available on request from the corresponding author. The data are not publicly available due to privacy or ethical restrictions.
